# Developing a Molecular Roadmap of Drug-Food Interactions

**DOI:** 10.1371/journal.pcbi.1004048

**Published:** 2015-02-10

**Authors:** Kasper Jensen, Yueqiong Ni, Gianni Panagiotou, Irene Kouskoumvekaki

**Affiliations:** 1 Department of Systems Biology, Technical University of Denmark, Kgs. Lyngby, Denmark; 2 School of Biological Sciences, The University of Hong Kong, Pokfulam, Hong Kong; University of Iowa, UNITED STATES

## Abstract

Recent research has demonstrated that consumption of food -especially fruits and vegetables- can alter the effects of drugs by interfering either with their pharmacokinetic or pharmacodynamic processes. Despite the recognition of such drug-food associations as an important element for successful therapeutic interventions, a systematic approach for identifying, predicting and preventing potential interactions between food and marketed or novel drugs is not yet available. The overall objective of this work was to sketch a comprehensive picture of the interference of ∼ 4,000 dietary components present in ∼1800 plant-based foods with the pharmacokinetics and pharmacodynamics processes of medicine, with the purpose of elucidating the molecular mechanisms involved. By employing a systems chemical biology approach that integrates data from the scientific literature and online databases, we gained a global view of the associations between diet and dietary molecules with drug targets, metabolic enzymes, drug transporters and carriers currently deposited in DrugBank. Moreover, we identified disease areas and drug targets that are most prone to the negative effects of drug-food interactions, showcasing a platform for making recommendations in relation to foods that should be avoided under certain medications. Lastly, by investigating the correlation of gene expression signatures of foods and drugs we were able to generate a completely novel drug-diet interactome map.

## Author Summary

Vegetables and fruits that are otherwise considered beneficial to our health can have serious consequences in medical care. Interference of plant-based foods with drug performance and pharmacological activity may potentially contribute to an increased risk of side effects or treatment failure. A well-known example of a drug-food interaction, the inhibitory effect of grapefruit juice on cytochrome P450, results in increased bioavailability of drugs such as felodipine, cyclosporine and saquinavir, which could lead to drug toxicity and poisoning. Although the importance of drug-food interactions has long been known, a systematic approach to identify, predict and prevent potential interactions between food and drugs has not yet been established. This work sets the ground for the understanding of the key molecular mechanisms of drug-food interactions with the scope to optimize therapeutic strategies and improve patient care. We tackle this problem using NutriChem, a database we have recently developed with information from the scientific literature and online databases related to natural compound origin and bioactivity. This systems chemical biology approach provides the basis for the identification and study of the substances in plant-based foods that affect the human proteins that are relevant for the pharmacokinetics and pharmacodynamics of current medicine.

## Introduction

Drugs and plant-based foods (i.e. fruits, vegetables and beverages derived from them, referred to simply as “food” throughout the rest of the document) manifest an intricate relationship in human health and have a complementary effect in disease prevention and therapy. In many diseases, such as hypertension, hyperlipidemia, and metabolic disorders, dietary interventions play a key part in the overall therapeutic strategy [1]. But there are also cases, where food can have a negative impact on drug therapy and constitute a significant problem in clinical practice. Recent research has demonstrated that foods are capable of altering the effects of drugs by interfering either with their pharmacokinetic or pharmacodynamic processes [2]. Pharmacokinetics includes the Absorption, Distribution, Metabolism and Excretion of drugs, commonly referred to jointly as ADME. Pharmacodynamic processes are related to the mechanisms of drug action, hence the therapeutic effect of drugs; interactions between food and drugs may inadvertently reduce or increase the drug therapeutic effect [3]. Until not long ago, our knowledge about drug-food interactions derived mostly from anecdotal experience, but recent scientific research can demonstrate examples, where food is shown to interfere with the pharmacokinetics and pharmacodynamics of drugs via a known, or partially known, mechanism of interaction: an inhibitory effect of grapefruit juice on Cytochrome P450 isoenzymes (e.g. CYP3A4) that leads to increased bioavailability of drugs e.g. felodipine, cyclosporin and saquinavir and potential symptomatic toxicity has been reported [4]; green tea reduces plasma concentrations of the β-blocker nadolol, possibly due to inhibition of the Organic Anion Transporter Polypeptide 1A2 (OATP1A2) [5]; activity and expression of P-glycoprotein (P-gp), an ATP-driven efflux pump with broad substrate specificity, can be affected by food phytochemicals, such as quercetin, bergamottin and catechins, which results in altered absorption and bioavailability of drugs that are Pgp substrates [6]; an antagonistic interaction of anticoagulant drug warfarin with vitamin K_1_ in green vegetables (e.g. broccoli, Brussels sprouts, kale, parsley, spinach), whereby the hypoprothrombinemic effect of warfarin is decreased and thromboembolic complications may develop [2]; sesame seeds have also been reported to negatively interfere with the tumor-inhibitory effect of Tamoxifen [7]. Judging from the examples above, under most *in vitro* drug-food interaction studies, food is either treated as one entity, or the study focuses on few, well-studied compounds, such as polyphenols, lipids and nutrients.

Our main hypothesis in the current work is that the interference of food on drug pharmacokinetic or pharmacodynamic processes is mainly exerted at the molecular level via natural compounds in food (i.e. phytochemicals) that are biologically active towards a wide range of proteins involved in drug ADME and drug action. The hypothesis is certainly supported by the large number of natural compounds that have reached the pharmacy shelves as marketed drugs. Hence, the more information we gather about these natural compounds, such as molecular structure, experimental and predicted bioactivity profile, the greater insight we will gain about the molecular mechanisms dictating drug-food interactions, which will help us identifying, predicting and preventing potential unwanted interactions between food and marketed or novel drugs. However, unlike drug bioactivity information that has already been made available for system-level analyses via databases such as ChEMBL (www.ebi.ac.uk/chembldb/) [8] and DrugBank (http://www.drugbank.ca/) [9], biological activity data and origin information of natural compounds are scarce and unstructured. To this end, we have developed a database generated by text mining of 21 million MEDLINE abstracts that pairs plant-based foods with the natural compounds they contain, their experimental bioactivity data and related human disease phenotypes [10,11]. In the present work, we are exploring this resource for links between the natural compound chemical-space of plant-based foods with the drug target space. By integrating protein-chemical interaction networks and gene expression signatures we provide a methodology for understanding mechanistically the effect of eating behaviors on therapeutic intervention strategies.

## Results

### The drug-like chemical space of plant-based diet

**Fig 1:**
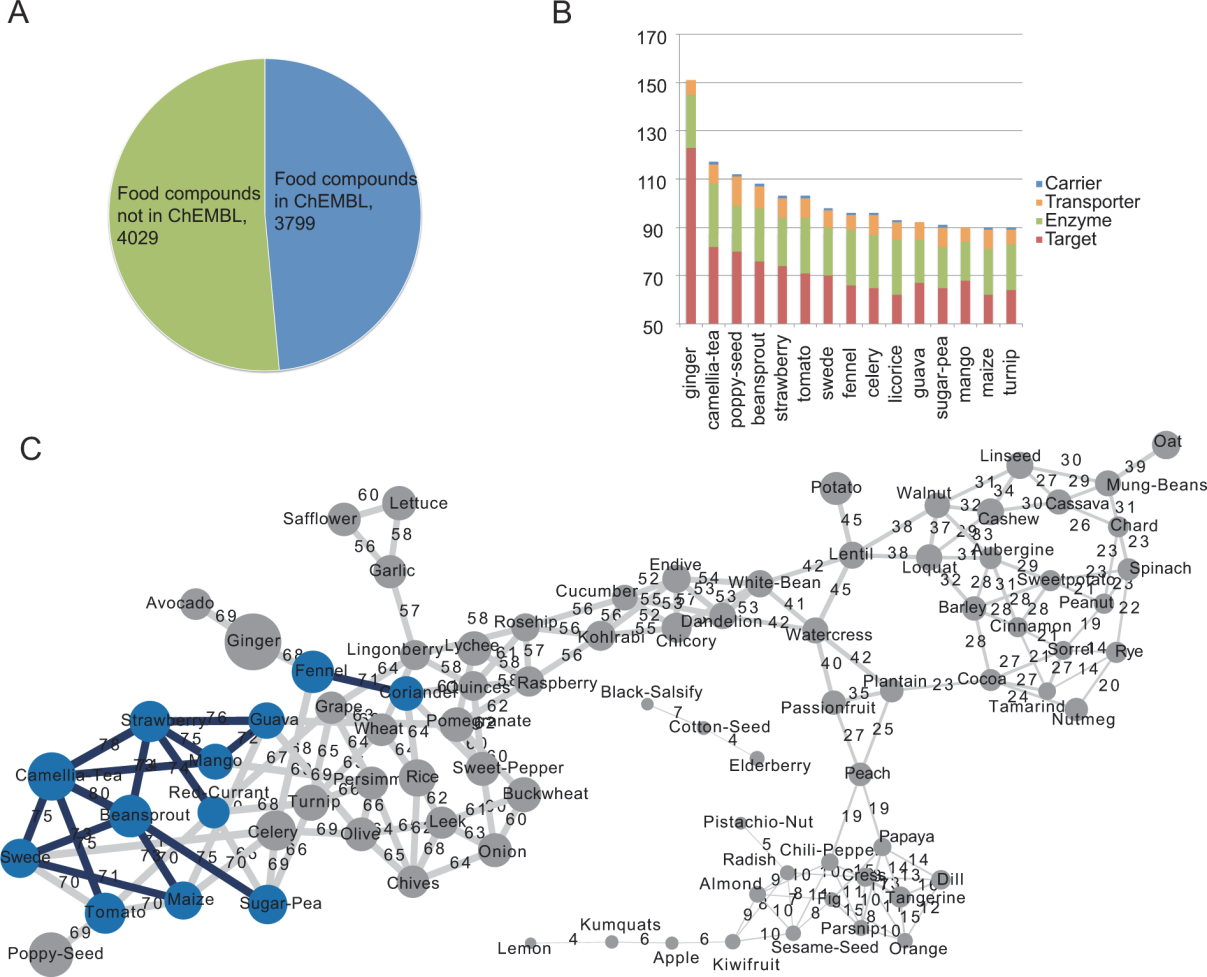
A molecular based view of drug-food interactions. **(A)** Number of plant-based food compounds in our database with (blue) and without (green) experimental bioactivity information in ChEMBL. **(B)** The plant-based foods with the most interactions to drug targets, carriers, transporters and enzymes. The plot shows the 15 most interacting foods within these 4 categories. **(C)** Network of foods that interact with the same drug target proteins. Node size reflects the number of bioactive compounds (phytochemicals) and interacting proteins for a given food. Edge width reflects the number of common interacting proteins between two foods. The nodes with the highest number of bioactive phytochemicals and interacting proteins are shown in blue. The edges with the highest number of common interacting proteins are shown in black. For visualization purposes, the top 5 edges for each node are shown on the network, while the complete data are provided as supplementary material (S1 Table).

In order to sculpt the chemical space of the natural compounds included in plant-based foods, we resorted to our recently developed resource NutriChem (www.cbs.dtu.dk/services/NutriChem-1.0) [11], which includes 1,772 plant-based foods associated by text-mining with ∼8,000 unique natural compounds (a.k.a. phytochemicals). Experimental bioactivity information exists in ChEMBL for less than half of these food compounds (Fig. 1A). Within this cluster, we identified 463 phytochemicals with bioactivity at the range of drug activity against 207 drug targets (i.e. targets related to drug pharmacodynamics), as well as 18 enzymes, 7 transporters and 3 carriers, ADME-relevant targets as deposited in DrugBank v.3. As shown in Fig. 1B, foods that are routinely part of our diet, such as strawberry, tomato, celery and maize, are involved via their bioactive phytochemicals in a high number of interactions with proteins within these 4 categories.

Ginger’s phytochemical profile appears as the most biologically active, interacting in total with 151 proteins, most of which associated with drug pharmacodynamics. This molecular level evidence of food-drug interactions is also in line with the information from the scientific literature assembled in NutriChem, which links ginger with 87 different human disease phenotypes. It should be pointed out that the 15 highly interacting foods shown in the figure are not necessarily the best characterized in terms of number of assigned phytochemicals. The number of bioactive phytochemicals in them ranges from 18 for mango to 42 for camellia-tea, while foods like licorice and rhubarb, for example, contain similar number of bioactive compounds (33 and 24 respectively) without, however, interacting with as many proteins within these 4 categories. Thus, the above result is not the outcome of data incompleteness biases in the scientific literature, but rather points towards specific structural characteristics of phytochemicals dictating drug-food interactions.

In order to further hone in the dietary habits that augment the impact on drug efficiency, we created a network that relies on the number of unique protein interactions shared between different foods. As shown in Fig. 1C several sub-networks of foods interact with the same protein space; a property that could be taken into account when drugs targeting these proteins are prescribed. For example, safflower, lettuce and garlic form a small sub-network that shares more than 55 proteins with experimental activity data involving their phytochemicals. The most broadly active food group consists of guava, mango, strawberry, beansprout, camellia-tea, swede and tomato, with the average number of shared interacting proteins being more than 70. Papaya, orange, dill, tangerine, cress and chili pepper, along with a few more foods, form an isolated module interacting with a separate protein target space. In all food clusters of Fig. 1C it is apparent that there is no phenotypic or higher level taxonomic characteristic of the foods that could be used to predict the shared interactions with the therapeutic protein space; this pattern has emerged from similarities in their phytochemical space.

### Effect of drug-food interactions on drug pharmacodynamics and pharmacokinetics

**Fig 2:**
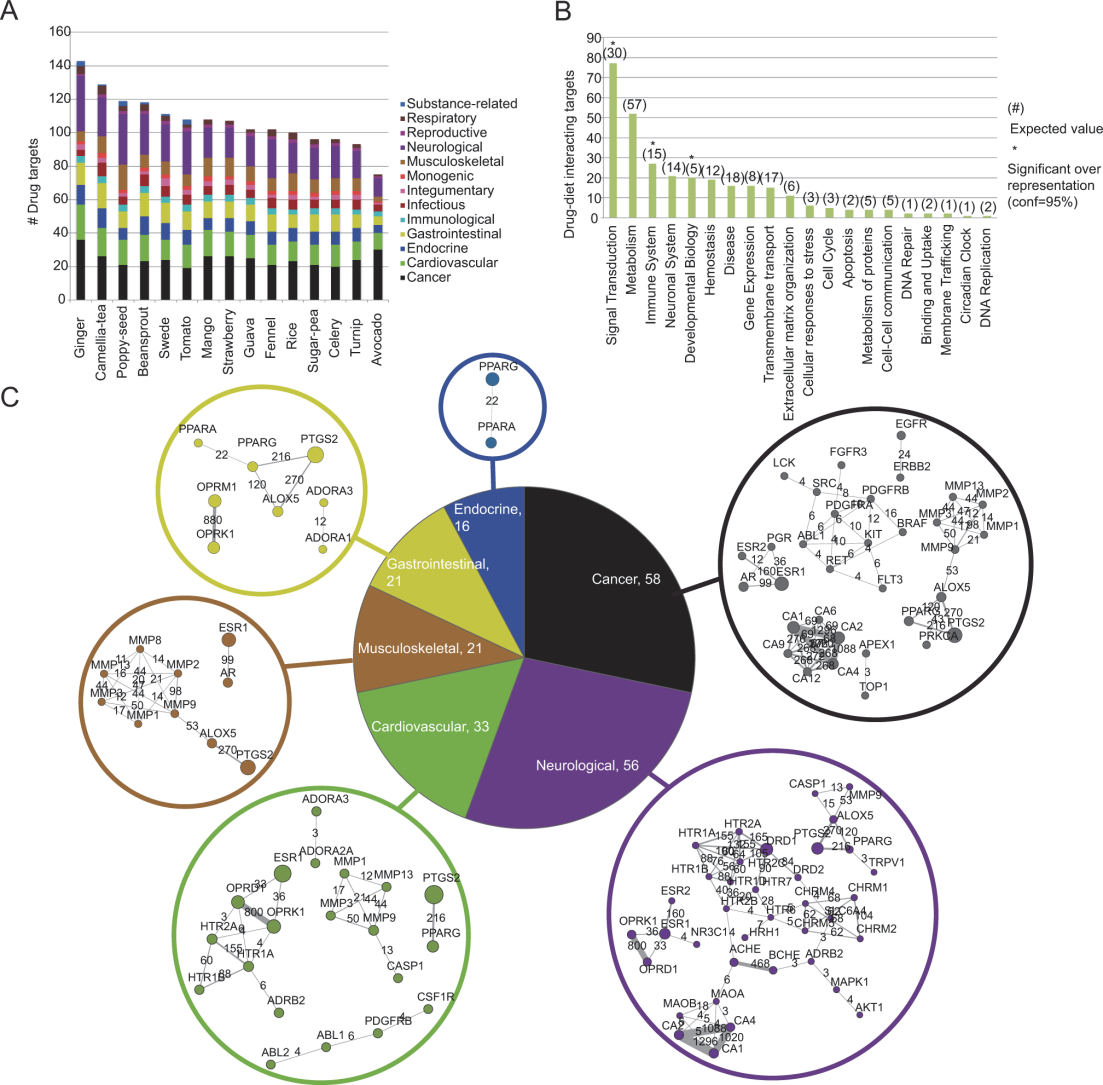
Drug-food interactions affecting drug pharmacodynamics. **(A)** The plot shows the plant-based foods with the highest number of interactions with drug targets and their associated human disease classification. **(B)** The number of drug targets affected by food, annotated to different biological systems. The expected number of targets in each biological category was calculated as: exp = (tpc / tdt) * tpa, where, tpc: the total number of drug targets from DrugBank in a biological category, tdt: the total number of drug targets in all biological categories (1,806 proteins) and tpa: the total number of drug targets that participate in drug-food interactions based on our analysis (186 proteins). **(C)** Networks of drug targets affected by food, per human disease class, shown for the 6 disease classes with the highest number of drug targets involved in food interactions. Two drug targets are connected when there are at least 3 drug-food pairs with biological activity against both proteins. The numbers inside the pie correspond to the total number of drug targets under each disease class that are affected by food. For visualization purposes, we show only the top 5 edges for each node, while the complete data are provided as supplementary material (S2 Table).

To get an insight of the pharmacodynamics processes that are mostly affected by the bioactive phytochemicals of our diet, we zoomed in on the interactions with drug targets. Comparing Fig. 2A, which presents the foods with the highest number of interactions with targets involved in drug pharmacodynamics, with Fig. 1B that relies on all protein interactions of a drug (target, transporter, carrier and metabolic enzyme), we notice that rice and avocado have replaced maize and licorice in the top-15 list. Furthermore, categorizing drug targets based on their human disease association, demonstrates the broad spectrum of disease treatments that may be affected by dietary habits. As shown in Fig. 2A, drug targets for 13 disease categories, ranging from cancer, neurological and cardiovascular to infectious and immunological diseases, could be potentially altered by food components. Another observation from Fig. 2A, not surprising due to the well-known protective role of plant-based diet against cardiovascular and gastrointestinal diseases, is that their drug targets are highly associated with phytochemical activity. Furthermore, looking into the association between food and drug targets at a biological process level reveals a wide range of functions that are targeted by food components (Fig. 2B). Nevertheless, our analysis points to that foods “show a preference” towards a specific drug target space that is significantly overrepresented (Student’s t-test, p<0.05) with proteins involved in signal transduction, immune system and developmental processes (Fig. 2B).

Having identified the foods that interact the most with drug targets and the biological processes that these targets participate in, we took a step further and zoomed into individual proteins. We constructed a network for each disease category (Fig. 2C), which links drug targets based on the drug-food pairs they share. For example in cancer, drug targets of the carbonic anhydrase family are tightly connected, as they share a large number of drug-food pairs. Looking into the neurological diseases we could identify a tight connection between the kappa- and delta- type opioid receptors, while for cardiovascular diseases a network of the 5-hydroxytryptamine receptors is highly targeted by the same drug-food pairs. Naturally, since many of the drug targets are shared between different disease classes, some of these networks were observed in more than one disease category. Nevertheless, similarly to our observations above at the biological process level, our analysis here revealed that drugs developed for certain protein targets are more prone to be affected by diet than others.

**Fig 3:**
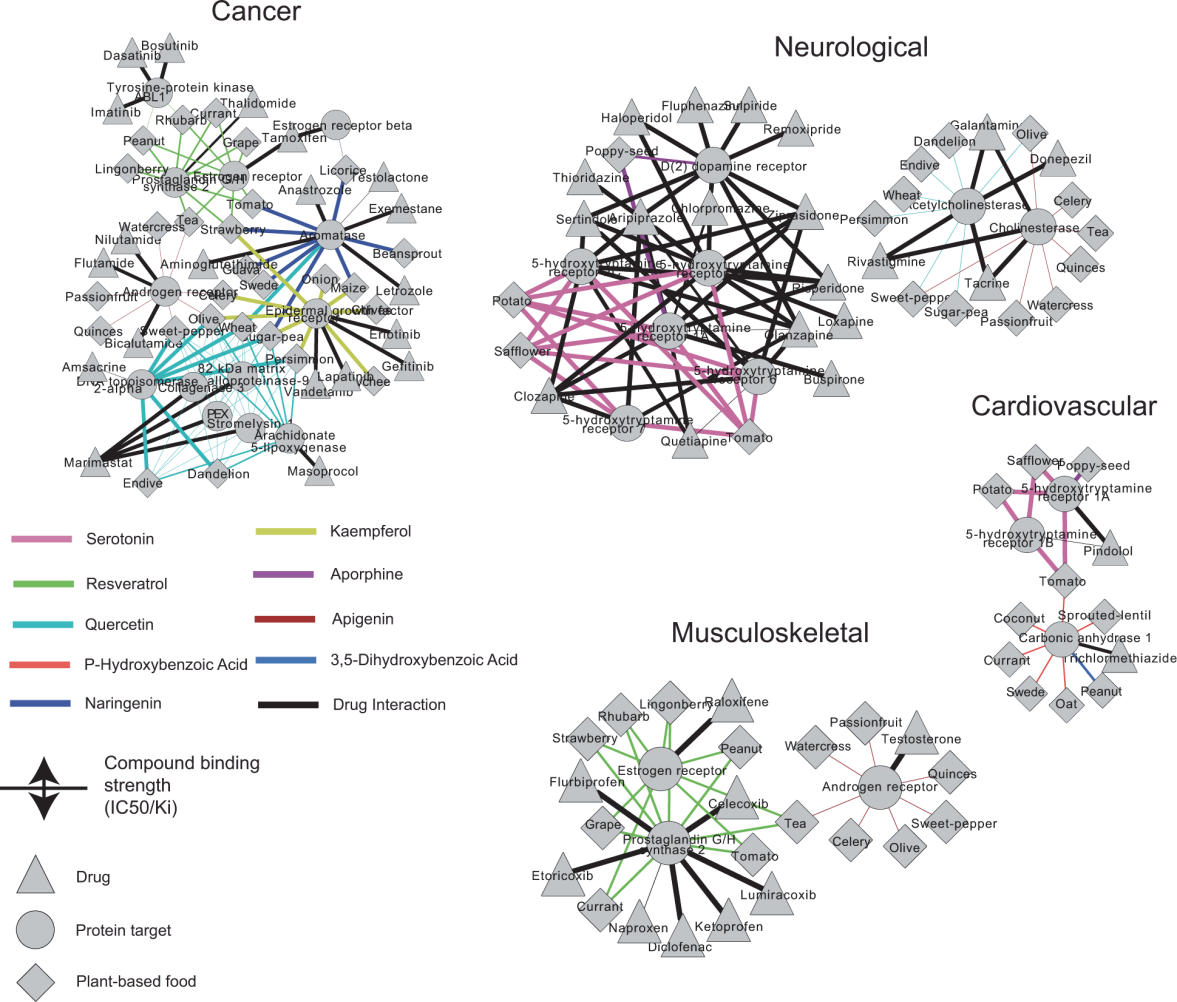
Disease-specific networks of drug-food interactions on proteins affecting drug pharmacodynamics. Node shape denotes drug target (circle), drug (triangle) and food (diamond). Edge color highlights the food compound (phytochemical) that shows the highest binding activity to the effect target. Edge width denotes the biological activity (Ki, IC50) and ranges between 1nM to 1000 nM. For visualization purposes, only 3 drugs and 3 foods with the highest biological activities are shown for each drug target, while the complete data are provided as supplementary material (S3 Table).

In order to explore the identified drug-food interactions at a molecular level, we selected case studies from 5 disease categories and highlighted the food components with the highest binding affinity to the drug targets. As shown in Fig. 3, aromatase, a protein targeted by 5 anticancer drugs (Anastrozole, Testolactone, Exemestane, Letrozole and Aminoglutethiumide) is also targeted by naringenin (IC50 = 2.9nM), a compound found in licorice, beansprout and maize, among others. Kaempferol, present in lychee, onion, strawberry and other common foods has a high binding affinity (IC50 = 3.0nM) for the epidermal growth factor receptor, target of Lapatinib, Gefitinib, Vandetanib and Erlotinib anticancer drugs. For neurological diseases, serotonin, present in sunflower, potato and tomato interacts strongly (Ki = 1.1nM) with the 5-hydroxytryptamine receptors targeted by several drugs (e.g. Loxapine, Buspirone, to name a few), whereas, aporphine, present in poppy-seed, binds to the D(2) dopamine receptor (Ki = 527nM), target of Ofremoxipride, Sulpiride and other drugs (Fig. 3). P-hydroxybenzoic acid, a compound naturally found in coconut, currant, sprouted lentil, swede and other foods, binds strongly (Ki = 920nM) to carbonic anhydrase, target of the cardiovascular drug Trichlormethiazide. Lastly, resveratrol, a compound that earned its prophylactic reputation against cardiovascular diseases due to its presence in red wine (represented by grape in Fig. 3), was found in our analysis to interfere with the activity of several drugs (Diclofenac, Raloxifene, etc.) that target either the estrogen receptor or prostaglandin G/H synthase 2 (involved in musculoskeletal diseases).

**Fig 4:**
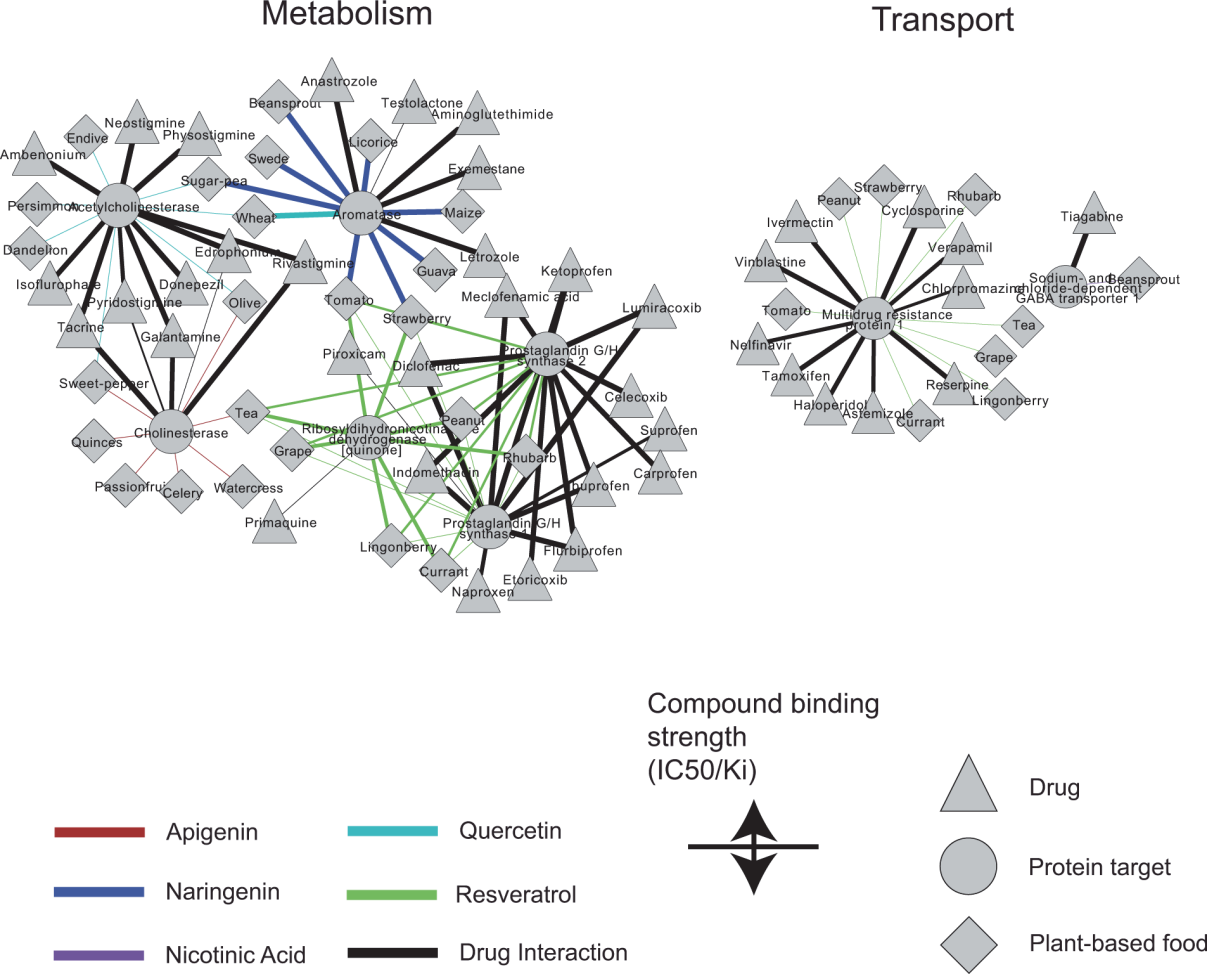
Networks of drug-food interactions affecting drug pharmacokinetics. Node shape denotes enzyme/transporter (circle), drug (triangle) and food (diamond). Edge color highlights the phytochemical with the highest binding activity to the protein target. Edge width denotes the biological activity (Ki, IC50) and ranges between 1nM to 1000 nM. For visualization purposes, only 3 drugs and 3 foods with the highest biological activities are shown for each protein target, while the complete data are provided as supplementary material (S4 Table).

Turning our focus to the effects of diet on the pharmacokinetics of drugs, we studied the interactions of phytochemicals with proteins involved in drug ADME. Fig. 4 illustrates the corresponding drug-food interaction networks for metabolic enzymes and transporters, where apigenin, quercetin, naringenin, resveratrol and nicotinic acid are the food components with the strongest binding affinities. These compounds are found in more than 40 foods; hence, a complete understanding of their interaction profile with drug ADME proteins is of utmost importance. For acetylcholinesterase and cholinesterase, two proteins involved in the metabolism of several drugs, e.g. Neostigmine (myasthenia gravis), Isoflurophate (glaucoma), Donepezil (dementia), Galantamine (dementia), the binding affinity of the phytochemicals quercetin and apigenin is lower than the one of the drugs, but still in the range of measured activities for these metabolic targets. In other cases, such as aromatase, involved in the metabolism of Anastrozole, Letrozole, Exemestane and Aminoglutethimide, drugs used against breast cancer, we encounter bioactive food components with stronger activities. Naringenin, a compound found in licorice, sugar pea, guava and others, has a binding affinity of IC50 = 1000nM against aromatase, comparable with the actual drug’s. Similarly, the ribosyl-dihydronicotinamide dehydrogenase, involved in the metabolism of primaquine, is targeted from resveratrol with binding affinity higher than that of the respective drug (IC50 = 450nM) (Fig. 4). Resveratrol interacts as well with the multidrug resistance protein 1, involved in the transport of several cancer drugs (Tamoxifen, Vinblastine) and other types of drugs, such as the antiretroviral drug Nelfinavir or Haloperidol an antipsychotic medication.

### Evaluation of drug-food interactions through their gene expression signatures

**Fig 5:**
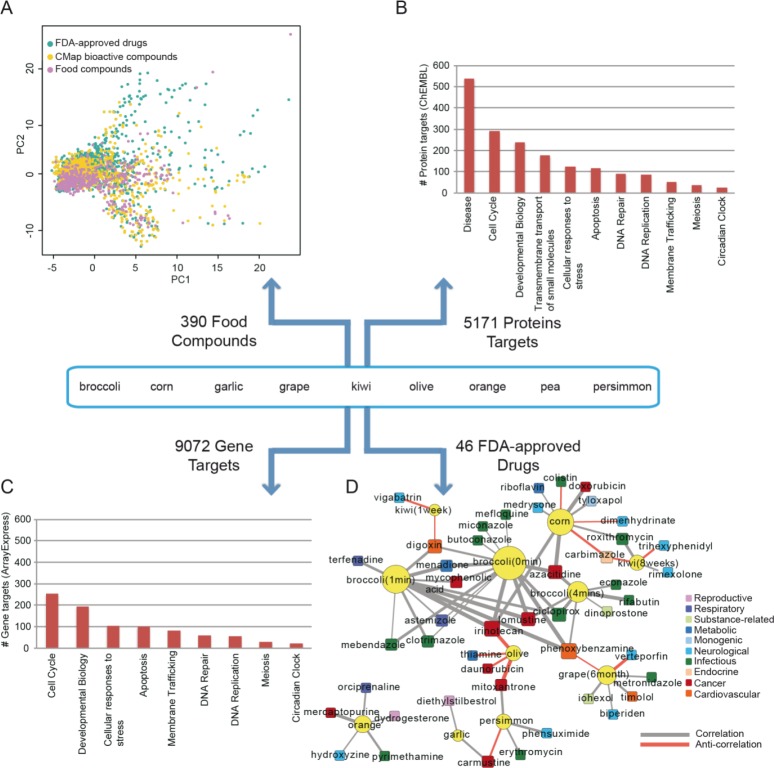
Comparative analysis of food and drug gene expression signatures. In total 9 high-quality gene expression signatures of plant-based foods could be retrieved (A) Chemical similarity comparison between 390 phytochemicals, the bioactive compounds present in ConnectivityMap (1,309) and FDA-approved drugs in DrugBank. (B) Pathway enrichment analysis of the proteins targeted (directly and indirectly, see Materials and Methods) by the phytochemicals. (C) Pathway enrichment analysis of the 9,072 genes that were found collectively significantly differentially expressed in the pair-wise comparisons of each food with the respective control. (D) Drug-food interactions based on the food gene expression signatures submitted to CMap. Food (yellow nodes) and drug (squares with different colors based on disease classification) are connected if they show a correlated (grey edge) or anti-correlated (orange edge) gene expression signature. The width of the edge indicates the significance level of the observed correlation.

Despite a thorough investigation of the interaction network formed by the bioactive compound space of diet and the drug activity space, the obtained results of possible drug-food interactions heavily rely on the available data related to the phytochemical content of food as well as the activity of these molecules on human proteins. To overcome the barrier of data incompleteness we compared the gene expression signatures of diet with the ones of FDA approved drugs, looking for correlated and anti-correlated profiles. The statistical analysis was performed using the Connectivity Map [12] which includes gene expression signatures from 1,309 compounds, both FDA-approved drugs and bioactive compounds. We retrieved gene expression data for 9 foods that are linked in NutriChem with 390 unique compounds; these 390 compounds are chemically similar to both CMap compounds as well as FDA approved drugs currently present in DrugBank (Fig. 5A). We could also retrieve 5,171 protein targets (direct and indirect) of these compounds, where “disease” is the most enriched pathway with 538 protein targets involved (Fig. 5B). Other significantly enriched pathways include cell cycle, developmental biology and apoptosis. Looking into the gene expression profiles of these 9 foods, we noticed that collectively 9,072 genes appear significantly differentially expressed (FDR corrected moderated t-test, p<0.05) between the control samples and the diet interventions. Interestingly, when these gene targets were further analyzed, the biological processes that were found significantly enriched have a high similarity with the protein space targeted by the phytochemicals (Fig. 5B vs. Fig. 5C). However, what we also observed was that from the 5,171 proteins targeted by the food components, only 2,653 were found in the significantly differentially expressed (DE) gene list. In our attempt to understand the reasons behind this discrepancy we selected a sub-set of 56 food phytochemicals that were targeting proteins from both groups; the ones that showed a significantly different expression level and the ones that did not (non-DE). We compared the binding affinity for those compounds having both DE targets and non-DE targets as well as the protein connectivity of their targets. While the protein connectivity analysis did not yield a statistically significant difference between the two groups, the IC50 values were significantly lower (Wilcoxon rank-sum test p value< 0.05) for the compounds targeting the non-DE group of genes.

Using as input the gene expression signatures of each of the 9 foods, we retrieved 133 CMap compounds, of which, 46 FDA approved drugs that have a significant correlated or anti-correlated profile (Fig. 5D). These 46 FDA approved drugs were further mapped to disease categories showing that mostly drugs used against infectious diseases (13), cancer (9) and neurological diseases (9) induce a gene expression signature that can be either enhanced or reversed by diet. In the drug-food interaction network shown in Fig. 5D, broccoli has the highest number of connections with drugs. Interestingly, all connections between broccoli and drugs are correlation-based, most of which display strong correlation coefficients. This finding sheds some additional light-from a mechanistic point of view- on the well-known beneficial effect of broccoli on human health. Orange and garlic induce a gene expression profile that is highly correlated with drugs used in cancer (Carmustine, Mercaptopurine) and reproductive disorders (Dydrogesterone, Diethylstilbestrol); orange specifically, is highly correlated with the activity of Orciprenaline (drug against a metabolic disease), Pyrimethamine (drug against an infectious disease) and Hydroxyzine (drug against a neurological disease). One notable case is olive oil; olive oil induces a gene expression signature highly anti-correlated with the anticancer drugs Mitoxanthrone, Irinotecan and Daunorubicin. Mitoxanthrone and Daunorubicin are typically used against leukemia, where olive oil has not demonstrated any beneficial effect. Irinotecan, on the other hand, is a drug used against colon cancer, a disease which several studies suggest that olive oil has actually a prophylactic effect on.

The analysis presented here should serve as a proof-of-concept comparison of the global gene expression responses induced by drugs and foods. The food gene expression signatures used here come from multiple research groups, diverse experimental designs and different tissues from animal models or human subjects, which may influence the correlation with the drugs. Nevertheless, the significant reduction of next generation sequencing cost is expected to positively influence nutritional studies as well, and allow transcriptome profiling of diets in a high throughput manner that could then be analyzed using our approach for possible interactions with drugs.

## Discussion

Plant-based therapies have been used for a variety of symptoms for thousands of years while recently there has been a drastic growth in the consumption of herbs and natural supplements with health benefits. In relation to AIDS and cancer patients especially, two life-threatening diseases where classical drug treatment does not always have a guaranteed effect, the use of both multiple prescription drugs and herbal supplements is very prevalent [13,14]. Given that components of herbs and natural supplements interact with human proteins in a similar manner as drugs, there is a high potential for altering drug efficiency. Furthermore, phytochemicals are abundant in our diet and have been shown *in vitro* to influence human proteins and cell-cultures. Several have demonstrated activity against the same proteins as drugs, and thus, potentially influence their pharmacokinetics and pharmacodynamics behavior when consumed concomitantly. In the example of sesame seed that has been reported to negatively interfere with the tumor-inhibitory effect of Tamoxifen [7], the protein responsible for the therapeutic effect of Tamoxifen is the estrogen receptor (P03372), which is also targeted by a number of different bioactive phytochemicals present in sesame, including beta-sitosterol [15][16]. Querying NutriChem for beta-sitosterol, we encounter it as phytochemical component of guava, onion, pomegranate, turnip, fennel, celery and kiwifruit—all common foods of our diet that could also be potentially involved in interactions with Tamoxifen and negatively affect its therapeutic activity. As another example, health professionals recommend to patients under medication against high blood pressure to avoid consumption of licorice (http://www.ehow.com/list_5798754_foods-avoid-taking-beta_blockers.html). The mechanism of this drug-food interaction is not yet clarified, although it has been occasionally attributed to the presence of glycyrrhizin. Our analysis points though towards the phytochemical liquiritin contained in licorice. Liquiritin has been found to interact with the beta-2 adrenergic receptor (P07550; Bioassay CHEMBL1738166), which is the primary target of beta-blockers, such as Penbutolol, a drug against hypertension.

The overall objective of this work was to gain knowledge on the interference of food phytochemicals with the pharmacokinetics and pharmacodynamics processes of medicine, with the purpose of elucidating the molecular mechanisms involved. To the best of our knowledge this is the first time of such a scale integration of data from the literature and online, publicly available databases coupled with gene expression analysis, for studying the effect of natural bioactive compounds from foods on proteins related to drug bioavailability and therapeutic effect. Our analysis brings into sight that cancer-related proteins are highly targeted by dietary molecules; since cancer is still one of the most deadly diseases, patients are willing to follow alternative therapeutic approaches, most often concomitantly with standard drug treatment, such as adopting a “healthy diet” that usually consists of fruits and vegetables. While this approach could be beneficial prior the onset of disease as a preventive measure, it should perhaps be adopted with caution when a patient is under drug therapy, as it may interfere with the therapeutic effect of the drug.

The novelty of the platform presented here is that it takes into account the global effects of food, propelled by its rich natural compound content, increasing the level of confidence of the scientific community and medical professionals when making recommendations for foods that should be avoided under certain medications. We illustrate that ignoring the complete phytochemical content of a food and focusing on a couple of “hot” molecules, a strategy widely applied in traditional food research, will never reveal the true magnitude of drug-food interactions. Furthermore, we identify clusters of foods that target the same therapeutic space as drugs, a property that could potentially increase the chances for severe alterations of the drug activity if these foods are consumed concomitantly. We also point out a large number of food components that are potentially involved in yet not documented drug-food interactions supporting the notion that ignoring the complete chemical content of a food is a missing link for obtaining a holistic view of the effect of diet. From a methodological point of view we believe that including the actual bioactivity values of the phytochemicals against proteins related to drug bioavailability and therapeutic effect allowed us to go beyond a simple enumeration of interactions to a more comprehensive and possibly accurate mapping of food-drug associations. Our food-drug interaction network reveals that therapeutic interventions for every disease category can be potentially affected to some degree by diet, even though specific disease areas, e.g. cancer and neurological diseases, are most prone to the negative effects of drug-food interactions than others. Lastly, we believe that we have demonstrated with several examples the power of a systems-level analysis to answering the two most important questions for patients and clinicians: (1) which foods should be potentially avoided from a patient under treatment, and (2) which is the underlying mechanism behind these drug-food interactions. However we should also keep in mind that, since many of the food compounds that are strong binders to proteins are very common in our diet, it will certainly be a daunting task to actually design diets that will not include any such compounds. Thus, adding in the network analysis the actual concentration in food of each bioactive compound would give a more accurate picture of the extent and severity of these drug-food interactions.

## Materials and Methods

### The food-drug interaction space

The plant-based food compounds and their chemical structures were retrieved from NutriChem 1.0 [11]. FDA-approved small molecule drugs were retrieved from DrugBank v.3 (http://drugbank.ca/ downloaded on Jan 12^th^, 2014). Food compounds and drugs were mapped to their protein interactions using ChEMBL v.16 (http://www.ebi.ac.uk/chembl/ downloaded on Sept 9^th^, 2013). Binding activities were retrieved from ChEMBL Bioassays. Protein targets were categorized into “Drug target”, “Enzyme”, “Transporter” and “Carrier”, following the DrugBank categorization. For a food compound to be considered active against a protein target, it has to bind within the range of the drugs targeting the same protein. For proteins, for which the binding activity of drugs was unknown, the binding activity of the food compound was compared to the mean value of the binding activities for proteins from the same category (i.e. drug target, enzyme, transporter or carrier). Drug protein targets were mapped to disease categories using the Therapeutic Target Database (http://bidd.nus.edu.sg/group/cjttd/ downloaded on Sept 9^th^, 2013) [17] and the Human Disease Ontology [18]. Disease categories were selected at the third level of the human disease ontology. Drug proteins were assigned to biological systems using Reactome (http://www.reactome.org).

### Gene expression signature comparison

**Chemical similarity between phytochemicals, CMap bioactive compounds and FDA-approved drugs.** SMILES strings of phytochemicals were retrieved from PubChem [19], while SMILES of the CMap bioactive compounds and FDA-approved small molecule drugs were retrieved from Connectivity Map build 02 [12] and DrugBank 3.0 [9], respectively. Based on the chemical structures, molecular and physical descriptors were calculated for each compound using the RDKit plugin (http://www.rdkit.org) in KNIME [20], including a 1024-bit Morgan circular fingerprint, Topological Polar Surface Area (TPSA), octanol/water partition coefficient (SlogP), Molecular Weight (MW), number of Lipinski hydrogen bond acceptors (HBA) and donors (HBD). Afterwards, a matrix of compound descriptors with 1029 columns was constructed, in which each row represented a phytochemical, a CMap bioactive compound, or an FDA-approved drug and a principle component analysis (PCA) using R was performed.

**Retrieval of direct and indirect protein targets of phytochemicals.** Phytochemicals were mapped to exact InChI key matches in ChEMBL and similar ChEMBL compounds using the Morgan circular fingerprint. The Tanimoto Coefficient (TC) was calculated based on Morgan fingerprint. Two compounds were similar if TC ≥0.85 and their difference in molecular weight lower than 50 g/mol. Next, the interactions of phytochemicals and proteins were annotated by searching in ChEMBL the protein targets of those exactly matched or similar ChEMBL compounds. The bioactivities were filtered based on the following thresholds: for K_i_, K_d_, IC_50_ and EC_50_, pchembl_value larger than 6; for inhibition, measurement value greater than 30%; for potency, measurement value lower than 50 μM. To deal with the multiple measurements of the same compound on the same protein, we calculated a frequency of “positive” measurements (served as evidence of compound-protein interaction) among all candidate measurements. Only chemical-protein interactions with a frequency of higher than 0.5 were considered confident and were used for further analysis. In addition to chemical-protein interactions from ChEMBL, we also included first-degree protein-protein interaction (PPI) partners (a confidence score higher than 400) from STRING 9.1 [21], in order to further expand our protein target space of phytochemicals. Note that this was only done for the protein targets of phytochemicals matching exactly to ChEMBL compounds. Only those PPI from human, rats and mice were included. After obtaining protein targets for phytochemicals, protein targets in rats and mice were mapped to their human orthologous proteins through Ensembl Biomart Homolog Service [22].

**Microarray data extraction and analysis.** ArrayExpress database [23] was first queried with a list of edible plants. Besides microarray data from human, experiments from rats and mice were included. For each microarray dataset, the raw data were background-corrected and normalized with RMA [24]. Quality check of the datasets was conducted in R with the arrayQualityMetrics package [25]. After manual inspection and quality check, 17 gene expression microarray experiments remained for downstream analysis. Differential expression analysis of pre-processed microarray data was performed with the R Bioconductor limma package [26]. A *p*-value of 0.05 after false discovery rate (FDR) correction for multiple hypothesis testing was used as the cutoff when selecting significantly differentially expressed (DE) genes [27]. Our analysis resulted in 9 foods with significant gene expression signatures and available phytochemical composition in NutriChem (see S5 Table). For each food, the list of DE genes was split into two lists of up- and down-regulated genes, referred to as “tag lists” in Connectivity Map [12]. The genes in the tag lists were converted to the CMap-compatible probe-set IDs from Affymetrix GeneChip Human Genome U133A Array. For DE genes in rats and mice, the human homolog genes were obtained through Ensembl Biomart Homolog Service [22] before mapping to required probe-set IDs. The paired tag lists were used to query Connectivity Map to reveal the correlation or anti-correlation relationship between foods and drugs. Based on the output from Connectivity Map, CMap drugs or bioactive compounds were considered to correlate or anti-correlate with foods if they had an absolute enrichment score higher than 0.75, permutation *p*-value less than 0.01 and a non-null percentage above 80%.

## Supporting Information

S1 Table Food network. Number of phytochemicals, total number of interacting proteins and number of common interacting proteins between two foods.
(XLSX)

S2 Table Drug target network. Number of shared drug-food interactions among drug targets under the same disease category.
(XLSX)

S3 Table Drug-food interactions affecting pharmacodynamics. Drugs and phytochemicals with activity against the same target protein.
(XLSX)

S4 Table Drug-food interactions affecting pharmacokinetics. Drugs and phytochemicals with activity against the same ADME-related protein.
(XLSX)

S5 Table Microarray Datasets. 9 foods with significant gene expression signatures and known phytochemical composition.
(XLSX)
